# Analysis of Tools Used in Assessing Technical Skills and Operative Competence in Trauma and Orthopaedic Surgical Training: A Systematic Review

**DOI:** 10.2106/JBJS.RVW.19.00167

**Published:** 2020-06-18

**Authors:** Hannah K. James, Anna W. Chapman, Giles T.R. Pattison, Joanne D. Fisher, Damian R. Griffin

**Affiliations:** 1 Clinical Trials Unit, Warwick Medical School, Coventry, United Kingdom; 2 Department of Trauma & Orthopedic Surgery, University Hospitals Coventry & Warwickshire, Coventry, United Kingdom

## Abstract

Robust assessment of skills acquisition and surgical performance during training is vital to ensuring operative competence among orthopaedic surgeons. A move to competency-based surgical training requires the use of tools that can assess surgical skills objectively and systematically. The aim of this systematic review was to describe the evidence for the utility of assessment tools used in evaluating operative performance in trauma and orthopaedic surgical training.

**Methods:**

We performed a comprehensive literature search of MEDLINE, Embase, and Google Scholar databases to June 2019. From eligible studies we abstracted data on study aim, assessment format (live theater or simulated setting), skills assessed, and tools or metrics used to assess surgical performance. The strengths, limitations, and psychometric properties of the assessment tools are reported on the basis of previously defined utility criteria.

**Results:**

One hundred and five studies published between 1990 and 2019 were included. Forty-two studies involved open orthopaedic surgical procedures, and 63 involved arthroscopy. The majority (85%) were used in the simulated environment. There was wide variation in the type of assessment tools in used, the strengths and weaknesses of which are assessor and setting-dependent.

**Conclusions:**

Current technical skills-assessment tools in trauma and orthopaedic surgery are largely procedure-specific and limited to research use in the simulated environment. An objective technical skills-assessment tool that is suitable for use in the live operative theater requires development and validation, to ensure proper competency-based assessment of surgical performance and readiness for unsupervised clinical practice.

**Clinical Relevance:**

Trainers and trainees can gain further insight into the technical skills assessment tools that they use in practice through the utility evidence provided.

Within an educational paradigm shift toward competency-based measures of performance in surgical training^1^, there is a need to evaluate surgical skills objectively and systematically, and hence, there is a drive toward developing more reliable and valid measures of surgical competence^1-3^.

Several surgical skill-assessment tools are currently in use in orthopaedic training, and studies evaluating the ability of these tools to objectively measure surgical performance have been performed. To our knowledge, this is the first systematic appraisal of the evidence for these assessment tools. It is imperative that the modernization of surgical curricula be supported by evidence-based tools for assessing technical skill and to enable summative judgments on progression through training and readiness for unsupervised operating.

The aim of this systematic review was to evaluate the orthopaedic surgical-competency literature and report on the metrics and tools used for skills assessment in trauma and orthopaedic surgical training; their utility with respect to validity, reliability, and impact on learning; and evidence for strengths and weaknesses of the various tools.

## Materials and Methods

This review was conducted in accordance with the Preferred Reporting Items for Systematic Reviews and Meta-Analyses (PRISMA) guidelines^4^ and registered with PROSPERO (International Prospective Register of Systematic Reviews)^5^.

### Data Sources

We performed a comprehensive literature search of MEDLINE, Embase, and Google Scholar electronic databases. The search strategy was developed by collating keywords from an initial scoping search (Table I). Categories 1, 2, and 3 were combined using Boolean “AND/OR” operators and results were limited to human subjects. No date or language limits were applied. The last search was performed in June 2019. Duplicates were removed, and retrieved titles were screened for initial eligibility.

**TABLE I:** Search Strategy

1. Competence.mp. OR assessment$.mp. OR skills$.mp. OR training.mp. OR performance.mp. 2. Technical.mp. OR operative.mp. OR simulation.mp. 3. Orthop$.mp. 4. Combine 1 AND 2 AND 3

### Study Selection

Eligible for inclusion were primary empirical research studies assessing postgraduate surgical resident performance in open or arthroscopic orthopaedic surgical skills in a simulated or live operative theater environment. Nonempirical studies and those that focused solely on patient or procedural outcome, or only described a training intervention, were excluded. A deliberately broad search strategy was employed to capture all studies in which an orthopaedic surgical skill was assessed.

### Title and Abstract Review

The search identified 2,305 citations. Initial title screening was undertaken by 1 author (H.K.J., a doctoral researcher), with studies that were obviously irrelevant excluded. One hundred and eighty-seven abstracts subsequently underwent screening by 2 authors (H.K.J. and A.W.C., an attending surgeon), and 106 were retrieved in full text. Of these, 105 were included in the final review (1 study was excluded at full-text review as the participants were not surgical residents). Studies were rejected at screening if they were not empirical research, if the study participants were undergraduates, or if nontechnical skills were being assessed; studies reporting simulator protocol development or validation were also excluded at this stage. The reference lists of full-text articles were examined for relevant studies, and those found by hand searching were subject to the same eligibility screening process.

### Data Extraction and Analysis

Data items relevant to the review objectives were extracted into a structured form to ensure consistency. The first reviewer undertook data extraction for all studies. Extracted data included study aim, setting, assessment format, number and training stage of participants, skills assessed, assessment tool and/or metrics, assessment tool category, study results, and “take-home” message related to the assessment tool. Assessment tools were classified by the type of method; the following categories were defined: traditional written assessments, objective assessment of technical skill, procedure-specific rating scale, individual procedural metrics, movement analysis, psychomotor testing, and subjective assessments.

## Results

### Search Results

**Fig. 1:**
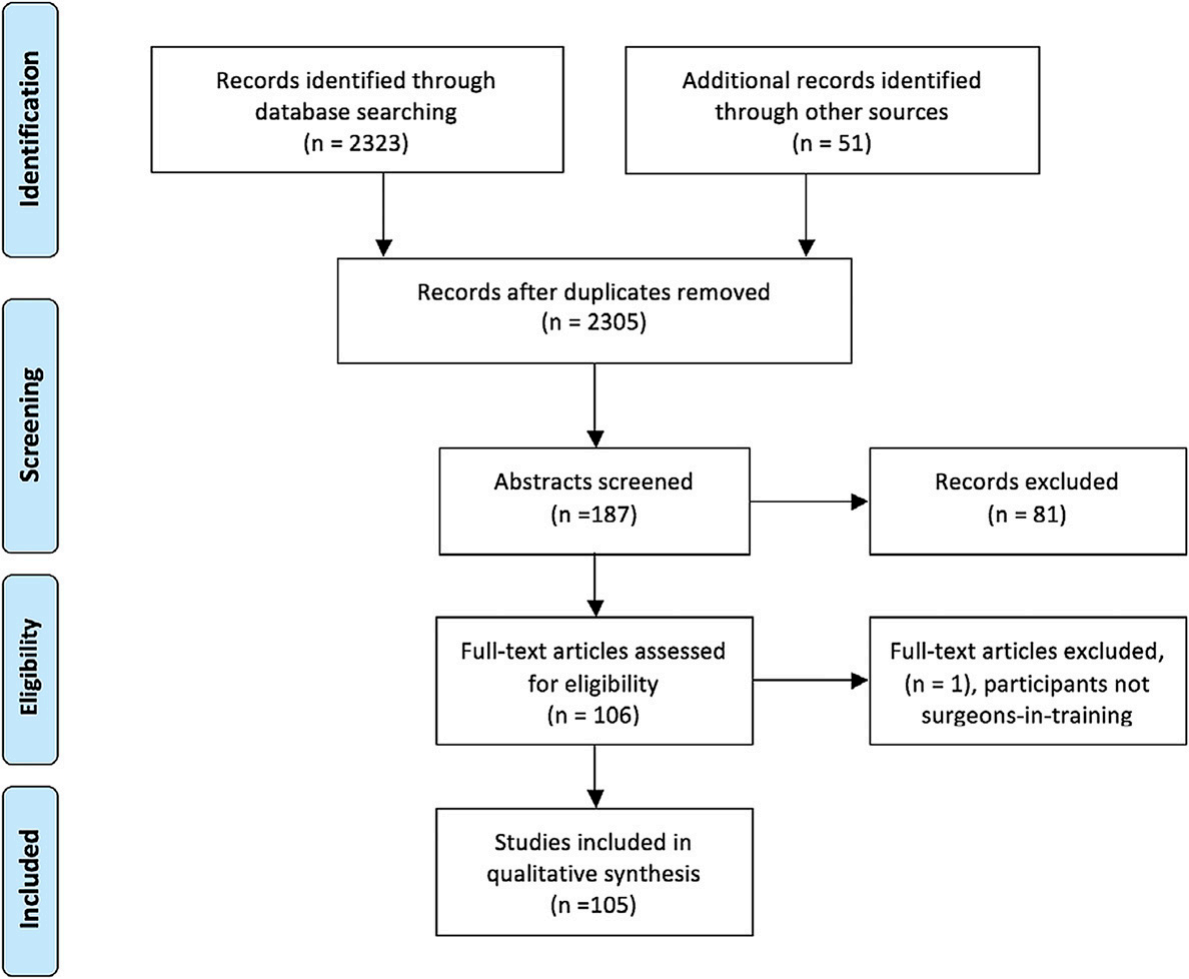
PRISMA flowchart.

One hundred and six articles were evaluated in detail, 1 of which was excluded at full-text review because the participants were not surgeons-in-training; 105 articles were therefore included in the review. The flow of studies is shown in Figure 1.

### Study Aims, Setting, and Participants

The studies were broadly split into 3 categories: studies measuring the impact of a simulation training intervention (26 studies^6-31^), studies assessing the construct validity of a simulator designed for training surgeons (42 studies^32-73^), and studies validating an assessment tool (37 studies^74-110^) (see Appendix Tables 1 and 2, column 1). Of the included studies, 60% assessed arthroscopic skill involving the knee (34 studies)^6,8,9,13,15,17,19,31-33,36,38,39,41,42,47,48,54-56,63,74,75,77-83,86,87,89,91^, the shoulder (25 studies)^7,12,15,16,18,20,37,40,44,45,49,51,53,54,56,63,76,77,81,82,88,90-92,110^, the hip (3 studies)^43,50,85^, the ankle (1 study)^14^, and basic general arthroscopic skills (6 studies)^10,11,34,52,57,84^. The majority (70%) of the studies assessing arthroscopic skill concerned diagnostic arthroscopy; procedural arthroscopic skills assessed included arthroscopic Bankart repair (3 studies), rotator cuff repair (1 study), labral repair (1 study), meniscal repair (2 studies), and anterior cruciate graft preparation (2 studies) and insertion (1 study) (see Appendix Table 1, column 5). The 42 studies that assessed open surgical procedures are shown in Appendix Table 2; as shown in column 5 of the table, the open procedures assessed included dynamic hip screw (DHS) fixation (4 studies), cannulated hip pinning (2 studies), and hemiarthroplasty (1 study) for a fractured femoral neck; spinal pedicle screw placement (6 studies); open surgical approaches to the shoulder (1 study); hand-trauma skills including nail-bed repair, Z-plasty, metacarpal fracture fixation, and tendon repair (1 study each); and various open reduction and internal fixation (ORIF) procedures for fractures of the forearm (7 studies), ankle (2 studies), and tibia (1 study), and complex articular fractures (1 study). Elective hand procedures, including trigger-finger release (1 study) and carpal tunnel decompression (3 studies), and elective hip (1 study) and knee (1 study) arthroplasty were also assessed.

The majority (85%) assessed skills in the simulated setting, 10 studies assessed skills in the live operative theater, and 10 studies assessed skills in both the simulated and live operative theater. Overall, 2,088 orthopaedic resident participants were involved in the studies, with experience level ranging from PGY (postgraduate year) 1 to 10.

### Assessment Format

The assessment format varied considerably (see Appendix Tables 1 and 2, column 3). Fifty-nine studies assessed performance using live observation, and 50 used post-hoc analysis of video footage by experts. Simulator-derived metrics were used in 72 studies. Final-product analysis by expert assessors was used for 3 studies, and biomechanical testing of the final product was used in 7.

### Assessment Tools or Metrics

A wide variety of assessment tools were used (see Appendix Table 3). Traditional assessments, such as written examinations, were used in 5 studies. Objective assessment of technical skills was widely used, and took many forms: task-specific checklists (20 studies), global rating scales (19 studies), and novel objective skills-assessment tools for both arthroscopy (22 studies) and open surgery (6 studies). Procedure-specific rating scales were used for both arthroscopic (7 studies) and open procedures (6 studies). Individual procedural metrics, such as final-product analysis and procedure time, were used in 56 studies. Movement analysis using simulator-derived metrics, such as hand movements, gaze tracking, hand-position checking, and instrument speed and path length, was used in 22 studies. Psychomotor testing using commercial dexterity tests was used in 5 studies. Subjective assessment measures were used in 4 studies.

### Quality Assessment

Van Der Vleuten described a series of utility criteria, known as the “utility index,” which is a widely accepted framework for assessing an evaluation instrument^111^. The features of the utility index are described in Table II. Each assessment tool was appraised for utility; the evidence for each of the various technical skills-assessment tools in current use is summarized according to the utility index criteria (see Appendix Table 3, columns 5 to 11). There was a wide spread of utility characteristics among the different tools, and their heterogeneity precludes any formal analysis. The strengths and limitations of the respective tools are presented in Appendix Table 3, columns 3 and 4.

**TABLE II:** Utility Criteria^111^ for Effective Assessment

Validity	The extent to which the skills claimed to be being assessed are assessed by the instrument
Content validity	Describes the appropriateness of the variables measured by the assessment instrument^122^
Construct validity	Describes the effectiveness of the assessment instrument at differentiating between different skill levels^122^
Concurrent validity	Describes the extent to which the assessment instrument agrees with existing performance measures^122^
Reliability	Describes the reproducibility of the results
Feasibility/acceptability	The extent to which the instrument is usable by the target audience
Educational impact	Consideration of the extent to which the instrument itself influences learning
Cost-effectiveness*	The extent to which the assessment instrument delivers value for money

* Not evaluated in this review.

## Discussion

Robust assessment of competency and operative skill in trauma and orthopaedic surgery is a topical issue in training. The primary goals of surgical-competency assessment are to provide a platform for learning through feedback, to make summative judgments about capability and progression through training, to maintain standards within the profession, and ultimately, to protect patients from incompetent surgeons^1^.

To our knowledge, this review is the first comprehensive analysis of the tools currently available for assessing technical skill and operative competency in trauma and orthopaedic surgical training.

The results show that none of the tools currently used for assessing technical skill in orthopaedic surgical training fulfill the criteria of Norcini et al. for effective assessment^112^. There is a similar deficiency of utility evidence in technical skills-assessment tools in general surgery^113^ and vascular surgery^1^, which face the same challenges as trauma and orthopaedics in moving toward a competency-based approach to training^1^.

Checklists and global rating scales were commonly used tools for technical skills assessment in the review studies (see Appendix Trable 3). Checklists deconstruct a task into discrete steps, and may have educational value for teaching procedural sequencing to novice residents. They do not capture the quality of performance, and the rigid binary scoring does not allow deviation resulting from there possibly being >1 acceptable way of undertaking a procedure. Another disadvantage of checklists is an early ceiling effect^1^. Checklists do have the advantage of being able to be administered by nonexpert assessors, and judgment on performance can be made either live or from video footage. They also can be used in both the simulated and live theater environment. They show reasonable construct validity^68,77,96,98^, concurrent validity^37,77,96,102,103^, and reliability^37,88,114^. With their limitations in mind, checklists are perhaps most appropriate for novice learners in a formative setting^1^.

Global rating scales use generic domains with a Likert-type scale and descriptive anchors to capture the quality of performance^61,66,93^. They are generalizable between procedures and can be used to assess complex procedures when there is >1 accepted method. They can discriminate between competent and expert performance, and there are many studies demonstrating their content^17,96^ and concurrent validity^17,77,85,96,98,103^ and their reliability^17,37,66,96^. They require expert surgeon evaluators and are more time-consuming to administer, and may be susceptible to assessor bias, as domains of assessment such as instrument handling and respect for tissue are inherently quite subjective. The ability of global rating scales to distinguish between all levels of performance and the absence of a ceiling effect make them useful for high-stakes, summative assessment^1^ and the assessment of advanced residents.

Several novel objective assessment tools have been developed and combine task-specific checklists with a global rating scale. The most promising front-runners among these are the Arthroscopic Surgical Skill Evaluation Tool (ASSET)^36,37,77^, which combines a task-specific checklist with an 8-domain global rating scale with end and middle descriptive anchors, and the Objective Structured Assessment of Technical Skills (OSATS) tool^23,93^ (see Appendix Table 3). While the ASSET is obviously restricted to arthroscopic procedures, both have a growing body of evidence across all domains of the utility index (Table II). The hybrid approach of combining a task-specific checklist and a global rating scale into 1 assessment tool enables the strengths of both to be brought together within a single tool but has the disadvantage of becoming long and burdensome to complete, which negatively impacts their feasibility and acceptability in a busy workplace in which training assessment conflicts with service pressures.

The OSATS tool is in current use in training programs in obstetrics/gynecology^115^ and ophthalmology^116^ and is popular with residents^117^. It captures the quality of performance and can distinguish competence from mastery, and the stages of progression in between. There were several studies in this review that demonstrated the validity, reliability, feasibility, and educational value of the OSATS tool in trauma and orthopaedics in the simulated setting (see Appendix Table 3, columns 5 to 11). Further work is required to assess its utility in the live operative theater.

There are a variety of procedure-specific rating scales that have been developed for both open^21,32,58,70,99,118^ and arthroscopic^7,76,81,82,90,92^ procedures (see Appendix Table 3). Most are in the early stages of validation and are likely to be most useful for the research setting. They are not practical for the live workplace environment given the variety of procedures that are undertaken within a typical training rotation; a generic tool that may be applied to the assessment of all procedures is more feasible.

Motion analysis (see Appendix Table 3) is also promising for assessing technical skill, particularly in arthroscopy, and several studies in this review demonstrated its utility^6,13,31,34,41,50,66,74,75,86^. Its use to date has been largely restricted to the research setting, and further work on transfer validity and potential educational impact is required. Some of the obvious barriers, such as sterility concerns, have been mitigated by using elbow instead of hand-mounted sensors in the live operative theater^31^. Hand-motion analysis can generate a sophisticated data profile that can detect subtle improvement in surgical performance, and may be able to measure the attainment of mastery. Other motion parameters, such as gaze tracking^6^, triangulation time^74^, instrument path length^12,15,40,48,49,51,55,56,63,110^, and collisions^38,55^, have demonstrated construct validity and feasibility in the simulated environment but are unlikely to be useful in the live operative theater, as most of these measurements are derived from the simulator itself.

Individual procedural metrics can also be used to assess technical skill (see Appendix Table 3). Final-product analysis provides an objective assessment of final product quality, from which technical proficiency is inferred. Examples include tip-apex distance in DHS fixation^58,62^, screw position^22,30,59,71,95^, and articular congruency^73,93^. Orthopaedics has the advantage of the routine use of intraoperative and postoperative radiographs from which relevant, real-life final-product analysis metrics such as implant position can easily be measured. Final-product analysis is objective and quite easy and efficient to perform. A nonspecialist assessor (who has been appropriately trained) can make the measurements. In the simulated setting, invasive final-product-analysis measures, such as biomechanical testing of a fracture construct, can be used to assess procedural success. Final-product analysis is appealing as it relates technical performance to real-world, clinically relevant measures of operative success. Conclusions regarding the construct validity of final-product analysis are, however, rather mixed, with almost as many studies refuting its construct validity^59,65,68,73,84^ as those demonstrating it^22,24,30,58,61,71,72,97^, and the studies analyzed did not demonstrate evidence of reliability.

Procedure time was extensively used as a procedural metric to assess technical skill in the included studies. It is easy to measure in both the simulated and in vivo setting. It relies on the intuitive assumption that speed equates to proficiency. This is potentially problematic, as extrinsic patient and staff factors beyond surgeons’ immediate control could influence procedure time, and it gives no indication of quality of performance; procedure time may be measured as fast because the surgeon was a masterfully efficient operator, but alternatively they may have rushed the procedure and been careless. The evidence for construct and concurrent validity for procedure time is mixed, with many studies showing it can discriminate between experience levels^6,11,12,30,31,33,34,40,43-45,47,48,50,51,53-56,61,63,64,67,78,86,110^, and performs well against other types of assessment^6,18,47,86^, with others showing it cannot^18,20,23,57,60,62,72,73,99,102^. Both final-product analysis and procedure time are therefore unlikely to be useful in isolation, but rather could be used as adjunctive measures of technical proficiency.

### Limitations

This review is limited to the assessment of technical skills in trauma and orthopaedic surgery; the assessment of nontechnical skills for surgeons was not considered in our analysis. Nontechnical skills are undoubtedly an essential dimension of surgical competence and are rightly beginning to receive attention in the surgical education literature^119^. The perfect technical skills-assessment tool is therefore never going to be usable in isolation to comprehensively assess competence, but rather should form a key part of a battery of evidence-based assessment tools.

### Implications and Recommendations

There is growing dissatisfaction with the current technical skills-assessment tools within the surgical education community^105,120^, and an increasingly urgent need to develop an evidence-based assessment tool that is generalizable to the broad range of technical and nontechnical skills in trauma and orthopaedic surgery, and that satisfies the utility criteria.

The Procedure Based Assessment, which is the current main assessment tool used for high-stakes assessment in the U.K. training system, is lengthy to complete, comprising 40 to 50 tick boxes and 12 free-text spaces^105^. It was initially implemented prior to any formal validation beyond an initial consensus-setting (Delphi) process to define the domains^105,121^. Several years after its introduction, a large, pan-surgical-specialty validation study was undertaken^109^, with a particular focus on demonstrating the reliability of the rating scales^105^. Within this study, orthopaedics appears underrepresented, with the totality of the procedure-based assessment-validity evidence relating to 2 orthopaedic procedures involving 7 residents. Subsequent validation work, using more traditional frameworks in general and vascular surgery, has demonstrated that the procedure-based assessment is a valid and reliable measure of performance^105^ and responsive to change^105^, but there remains a deficiency of evidence for its utility in orthopaedics, which is surprising given that it is the current gold-standard assessment in the U.K. training system (see Appendix Table 3). Adding to the problem, engagement with the Procedure Based Assessment has been poor^105^, and it remains unpopular^120^. A national survey of trauma and orthopaedic resident attitudes toward procedure-based assessments (PBAs) in the U.K. found that more than half agreed or strongly agreed with the statement “completing PBAs is nothing but a form-filling exercise,” 60% agreed or strongly agreed that there are “barriers to the successful use of PBAs by residents”^120^, and only one-third believed that they should be used for high-stakes assessment in training, such as the Annual Review of Competence Progression^120^. Further work has found that reasons for the poor engagement are that the Procedure Based Assessment is burdensome to complete; with a coarse rating scale of blunt, binary descriptors, it cannot distinguish mastery or higher-order skills; and it results in general assessment fatigue^105^.

The Procedure Based Assessment was among the earliest formal tools for technical skills assessment in orthopaedic surgical training, and its creators deserve recognition for beginning the process of objectively assessing technical skills of surgeons-in-training. We propose that the Procedure Based Assessment is no longer appropriate for use in summative assessment in a modern competency-based assessment training environment. The OSATS tool and the ASSET show promise as replacements to the Procedure Based Assessment, and validation work on these, with a particular focus on their use in the live operative theater, should be continued.

### Conclusions

The evidence for the utility of the technical skills-assessment tools currently used in trauma and orthopaedic surgical training is inadequate to support their use in summative high-stakes assessment of competency. An assessment tool that is generalizable to the broad range of technical and nontechnical skills relevant to trauma and orthopaedics, that satisfies the utility criteria, and that is cost-effective and feasible requires development.

## Appendix

Supporting material provided by the authors is posted with the online version of this article as a data supplement at jbjs.org (http://links.lww.com/JBJSREV/A611).
